# High-titer production of 13*R*-manoyl oxide in metabolically engineered *Saccharomyces cerevisiae*

**DOI:** 10.1186/s12934-019-1123-z

**Published:** 2019-04-24

**Authors:** Chuanbo Zhang, Haiyan Ju, Chun-Zhe Lu, Fanglong Zhao, Jingjing Liu, Xiaoyan Guo, Yufen Wu, Guang-Rong Zhao, Wenyu Lu

**Affiliations:** 1 School of Chemical Engineering and Technology, Tianjin University, Tianjin, 300350 People’s Republic of China; 2 Key Laboratory of System Bioengineering (Tianjin University), Ministry of Education, Tianjin, 300350 People’s Republic of China; 3 SynBio Research Platform, Collaborative Innovation Center of Chemical Science and Engineering (Tianjin), Tianjin, 300350 People’s Republic of China

**Keywords:** 13*R*-manoyl oxide, Diterpene, Metabolic engineering, *Saccharomyces cerevisiae*

## Abstract

**Background:**

Diterpenoids are a large class of natural products with complex structures and broad commercial applications as food additives, important medicines, and fragrances. However, their low abundance in plants and high structural complexity limit their applications. Therefore, it is important to create an efficient diterpenoid-producing yeast cell factory of the production of various high-value diterpenoid compounds in a cost-effective manner

**Results:**

In this study, 13*R*-manoyl oxide (13*R*-MO; 2.31 mg/L) was produced by expressing *CfTPS2* and *CfTPS3* from *Coleus* *forskohlii* in *Saccharomyces cerevisiae*. The 13*R*-MO titer was increased by 142-fold to 328.15 mg/L via the stepwise metabolic engineering of the original strain, including the overexpression of the rate-limiting genes (*tHMG1* and *ERG20*) of the mevalonate pathway, transcription and protein level regulation of *ERG9*, Bts1p and Erg20^F96C^p fusion, and the overexpression of *tCfTPS2* and *tCfTPS3* (excision of the N-terminal plastid transit peptide sequences of *CfTPS2* and *CfTPS3*). The final titer of 13*R*-MO reached up to 3 g/L by fed-batch fermentation in a 5 L bioreactor.

**Conclusions:**

In this study, an efficient 13*R*-MO yeast cell factory was constructed, which achieved the de novo production of 3 g/L of 13R-MO from glucose. To the best of our knowledge, this is the highest 13*R*-MO titer reported to date. Furthermore, the metabolic engineering strategies presented here could be used to produce other valuable diterpenoid compounds in yeast.

**Electronic supplementary material:**

The online version of this article (10.1186/s12934-019-1123-z) contains supplementary material, which is available to authorized users.

## Background

Plants have evolved to produce diverse secondary metabolites in order to adapt to their living environment and protect themselves [1]. Terpenoids are the main components of plant secondary metabolites, some of which are important to humans as pharmaceuticals and biofuels [2, 3]. In the field of medicine, many studies have been performed on plant-derived terpenoid pharmaceuticals, such as the anticancer drug taxol [4] and anti-malarial drug artemisinin [5, 6]. Examples of plant-derived terpenoid biofuels include the jet air–fuel additives pinane, carane, and sabinane, monoterpenoids [3], and isoprenoid alcohols [7].

Forskolin, a labdane-type diterpenoid, was discovered in the roots of the medicinal shrub *Coleus* *forskohlii* and has been used to treat hypertension, heart complications, and asthma [8]. Forskolin has also been used as a cyclic AMP booster to treat glaucoma, hypertension, and heart failure [9]. NKH477, a semi-synthetic forskolin derivative, has been used commercially to treat surgical complications, heart failure, and cerebral vasospasm [10]. However, the complete synthesis of forskolin has not yet been achieved due to its stereospecific structure [11]. Recently, 13*R*-manoyl oxide (13*R*-MO), which was found in the root cork cells of *C.* *forskohlii*, was found to be the precursor of forskolin [12]. By mining root transcriptome data of *C.* *forskohlii*, CfTPS2 which synthesizes the intermediate copal-8-ol diphosphate from GGPP and CfTPS3 which catalyzes the stereospecific formation of 13*R*-manoyl oxide were confirmed to stereospecifically biosynthesize 13*R*-MO from geranylgeranyl diphosphate (GGPP) [12]. The large-scale production of forskolin is challenging due to its complex structure and low abundance in the native host [8]; however, when compared with chemical synthesis and plant extraction, forskolin biosynthesis is a feasible approach for commercial applications [11, 12].

Yeast is a particularly good host for terpenoid production as it has a native mevalonate (MVA) pathway and is robust for industrial fermentation and genetic engineering [13]. In *Saccharomyces cerevisiae*, acetyl-CoA is sequentially catalyzed by seven MVA pathway enzymes to produce isopentenyl diphosphate (IPP) and dimethylallyl diphosphate (DMAPP), which are the two five-carbon building blocks required for the synthesis of all terpenoids. Erg20p then catalyzes the condensation of IPP and DMAPP to produce the monoterpenoid and sesquiterpenoid precursors geranyl diphosphate (GPP) and farnesyl diphosphate (FPP), respectively [14]. GGPP is a diterpenoid precursor, derived from FPP and IPP via Bts1p catalysis [15]. A yeast strain that produces high titers (40 g/L) of amorphadiene has demonstrated potential for natural terpenoid production [16]; however, the yields of diterpene were markedly lower than that of sesquiterpenoids. Metabolic engineering to increase the FPP pool, such as increasing precursor levels, inhibiting the ergosterol synthetic pathway, and deleting branch pathways, was shown to remarkably increased sesquiterpenoid production [17, 18]. However, *S. cerevisiae* GGPP synthase (Bts1p) showed a higher *K*_*m*_ value (3.2 μM) for FPP than for squalene synthase (2.5 μM) [19]. Therefore, conversion of FPP to GGPP seems to be the rate-limiting step for diterpenoid production [20]. Many studies have attempted to increase the GGPP pool in order to increase diterpenoid production, for example, by overexpressing GGPP synthase [21], converting yeast endogenous Erg20p to GGPP synthase [20], fusing Bts1p and Erg20p [22], and repressing *ERG9* expression with a *P*_*MET3*_ promoter [23]. These efforts have made a significant contribution to increasing the production of diterpenoids in *S. cerevisiae*.

**Fig. 1:**
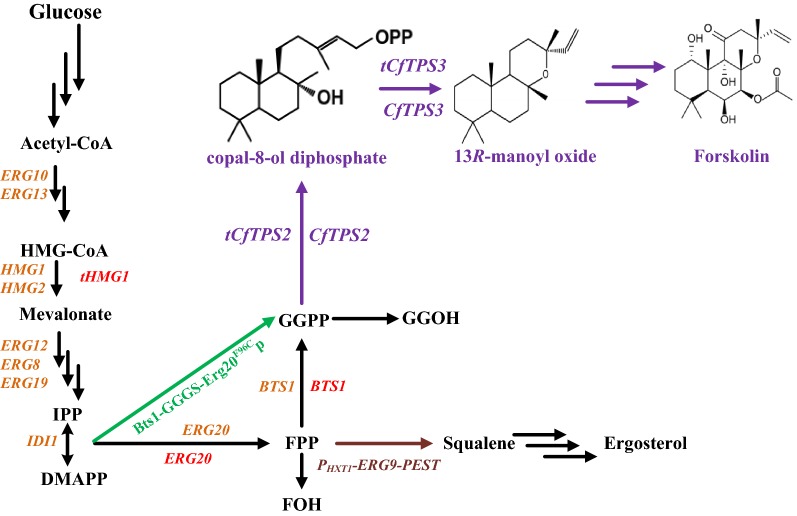
Schematic overview of the 13*R*-MO synthesis pathway in *S. cerevisiae*. Heterologous genes are marked in purple, native MVA pathway genes are marked in orange, overexpressed genes are marked in red are, and *ERG9* was downregulated as shown in kermesinus. The Bts1p and Erg20^F96C^p fusion protein is indicated in green. *Acetyl-CoA* acetyl coenzyme A, *HMG-CoA* 3-hydroxy-3-methylglutaryl-CoA, *tHMG1* truncated HMG-CoA reductase gene, *IPP* isopentenyl pyrophosphate, *DMAPP* dimethylallyl pyrophosphate, *ERG20* farnesyl diphosphate synthetase gene, *FPP* farnesyl diphosphate synthetase, *FOH* farnesol, *ERG9* squalene synthetase gene, *GGPP* geranylgeranyl diphosphate, *GGOH* geranylgeraniol

When an exogenous pathway is introduced into *S. cerevisiae*, regulating the overall metabolic pathway, and not just the rate-limiting steps, is important for maximizing the synthesis potential of the target product [24]. In this study, *CfTPS2* and *CfTPS3* were expressed in *S. cerevisiae* W303-1a, and 13*R*-MO was detected (2.31 mg/L). The 13R-MO titer was increased by 142-fold to 328.15 mg/L via regulation of the key genes of the MVA pathway and truncation of diterpene synthase (Fig. 1). The titer was further increased to 3001.46 mg/L by fed-batch fermentation.

## Results

### Engineering a 13*R*-MO synthetic pathway in *S. cerevisiae*

**Fig. 2:**
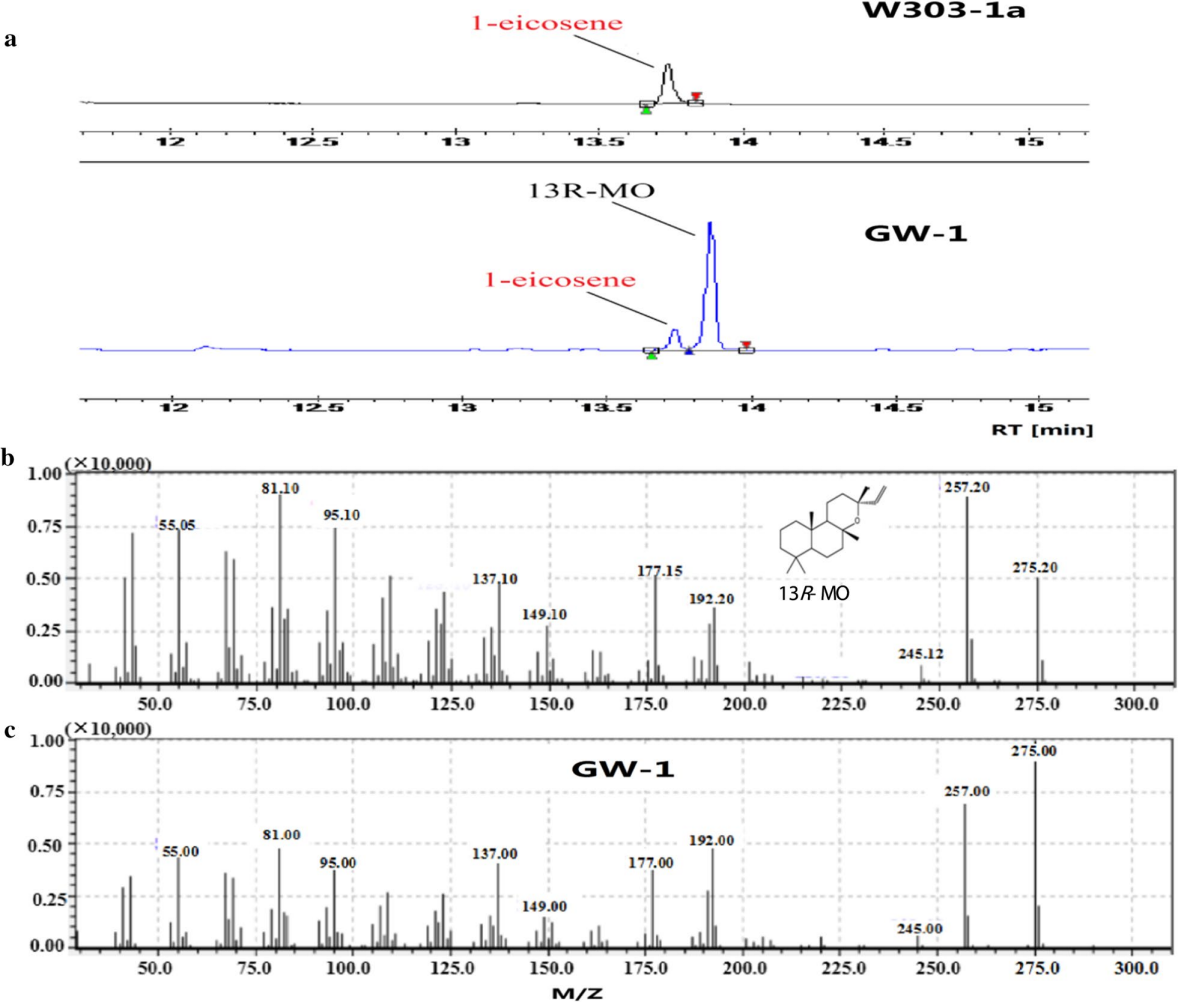
13*R*-MO production and identification in *S. cerevisiae*. **a** The chromatogram for 13*R*-MO production by strain GW-1 and the original strain W303-1a. **b** GC–MS spectra for 13*R*-MO reported in the literature [25, 26]. **c** GC–MS spectra of the chromatographic peak at RT = 13.86 min

In *S. cerevisiae*, the diterpenoid precursor GGPP is synthesized from the condensation of FPP and IPP by Bts1p. Two 13*R*-MO synthesis genes from *C.* *forskohlii*, *CfTPS2* and *CfTPS3*, were codon-optimized and integrated into the *ura3* site of *S. cerevisiae* W303-1a under the strong promoters *PGK1p* and *TDH3p* respectively, resulting in the GW-1 strain. As shown in Fig. 2, a new peak (RT = 13.86 min) was identified as 13*R*-MO based on previously reported mass spectra in the literature [25, 26]. Strain GW-1 could produce 2.31 mg/L of 13*R*-MO, which was quantified by comparing its peak area with the internal standard 1-eicosene, via 4 days cultivation in YPD medium.

### MVA pathway engineering to increase precursor supply for efficient 13*R*-MO biosynthesis

FPP and GGPP are the two crucial precursors for diterpenoid production in *S. cerevisiae.* Firstly, FPP supply was optimized to improve the production of 13*R*-MO. *CfTPS2* and *CfTPS3* were introduced into the well-established chassis strain LW03 (*tHMG1* and *ERG20* overexpressed and the *ERG9* promoter replaced by *MET3p*), which was constructed for the production of the sesquiterpene α-humulene in our previous study, resulting in strain LZJ1 [18]. LZJ1 could produce 2.86 mg/L of 13*R*-MO, which was slightly higher than that produced by the original strain GW-1. In order to reduce squalene accumulation, the strain LZJ2 was constructed by fusing a PEST (rich in Pro, Glu/Asp, Ser, and Thr) sequence [27] to the C-terminal of Erg9p to accelerate its degradation. The strain LZJ2 produced 3.52 mg/L of 13*R*-MO, which was 1.5-fold higher than that produced by the original strain GW-1.

GGPP production was then optimized in the *S. cerevisiae* strain LZJ2*.* The co-expression of *BTS1* and *ERG20*^*F96C*^ at the *HO* site of strain LZJ2 resulted in the strain LZJ3, which produced 7.78 mg/L of 13*R*-MO. A GGGS linker was then used to fuse Bts1p with Erg20p or Erg20^F96C^p in LZJ2, resulting in strains LZJ4 and LZJ5, which were able to produce 9.75 mg/L and 23.31 mg/L of 13*R*-MO, respectively.

### CfTPS2 and CfTPS3 truncation to enhance 13*R*-MO production

**Fig. 3:**
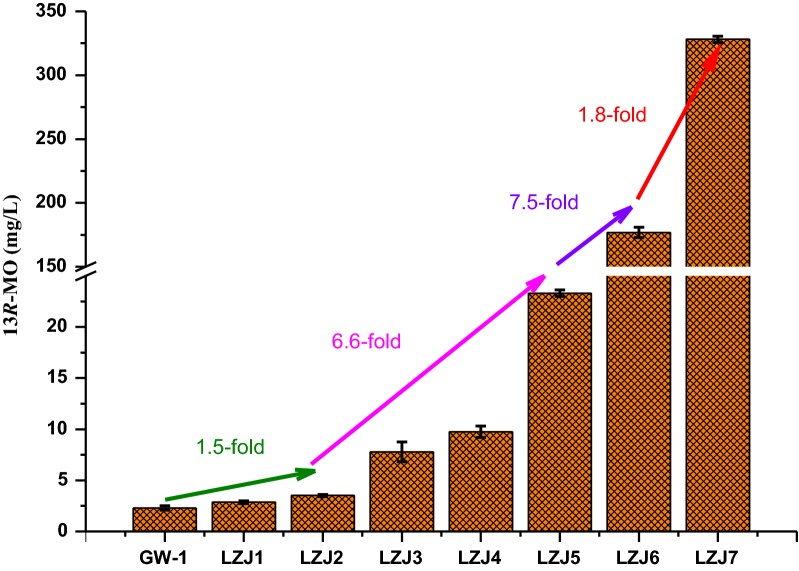
Stepwise increase in the production of 13*R*-MO in *S. cerevisiae.* YPD medium containing 20 g/L of glucose was used for fermentation. Error bars represent the standard deviation of three independent experiments

To generate pseudo-mature variants, the N-terminal plastid transit peptides of CfTPS2 and CfTPS3 were predicted using ChloroP (https://www.cbs.dtu.dk/services/ChloroP/) software and were removed to obtain tCfTPS2 and tCfTPS3. The coding sequences of the plastid transit peptides are shown in Additional file 1: Table S2. *tCfTPS2* and *tCfTPS3* were co-expressed in the *δ* sites of strain LZJ5 under the strong promoters *TEF1p* and *TDH3p*, respectively. The resultant strain LZJ6 could produce 176.8 mg/L of 13*R*-MO, which was 6.6-fold higher than that produced by its parental strain. The key rate-limiting enzymes of the MVA pathway, tHmg1p and Bts1-GGGS-Erg20^F96C^p, were over-expressed by the rDNA site insertion to obtain strain LZJ7, further increasing the production of 13*R*-MO to 328.15 mg/L. The stepwise increase in the production of 13*R*-MO is shown in Fig. 3.

The performance of each engineered 13*R*-MO production strain was evaluated in shake flasks by biphasic cultivation. The biomass, 13*R*-MO, farnesol (FOH) and geranylgeraniol (GGOH) content are listed in Table 1. A slight accumulation of FOH was observed in the original strain GW-1 (0.37 mg/L), and its production was increased in a stepwise manner by the regulation of key genes (*tHMG1*, *ERG20*, *BTS1* and *ERG9*) in the MVA pathway, with the highest titer (33.62 mg/L) detected in the strain LZJ3. GGOH also gradually accumulated to 40.91 mg/L via the stepwise MVA pathway engineering. Bts1-GGGS-Erg20p/Erg20^F96C^p fusion (strains LZJ4 and LZJ5, respectively) successfully drew FPP into the GGPP pool, and the highest GGOH accumulation, 93.12 mg/L, was detected in strain LZJ5. The N-terminal truncation of CfTPS2 and CfTPS3 in LZJ6 increased the 13*R*-MO titer by 7.5-fold compared to LZJ5, and decreased the GGOH titer to 51.13 mg/L from 93.12 mg/L, and decreased the FOH titer from 15.2 to 5.54 mg/L. The accumulation of GGOH and FOH were further decreased in the final strain LZJ7 (Table 1).

**Table 1:** Performance of the engineered strains in shake-flask cultivation

Strains	Biomass (mg/L)	13*R*-MO (mg/L)	GGOH (mg/L)	FOH (mg/L)
GW-1	6693.21 ± 88.56	2.31 ± 0.11	1.25 ± 0.18	0.37 ± 0.04
LZJ1	6762.8 ± 169.07	2.86 ± 0.13	6.56 ± 1.84	5.96 ± 0.67
LZJ2	6803.47 ± 102.96	3.52 ± 0.11	12.32 ± 0.69	20.11 ± 1.69
LZJ3	5575.93 ± 231.8	7.78 ± 0.97	33.91 ± 1.01	33.62 ± 4.39
LZJ4	6458.1 ± 147.06	9.75 ± 0.56	42.86 ± 2.23	16.33 ± 2.35
LZJ5	4968.76 ± 168.27	23.31 ± 0.31	93.12 ± 4.92	15.2 ± 0.79
LZJ6	5956.45 ± 133.06	176.8 ± 4.09	51.13 ± 0.61	5.54 ± 1.27
LZJ7	7344.7 ± 260.29	328.15 ± 2.56	17.32 ± 0.15	4.13 ± 0.17

“ ± ” represent the standard deviation of three independent experiments

### Batch and fed-batch fermentation of LZJ7 in a 5 L bioreactor

**Fig. 4:**
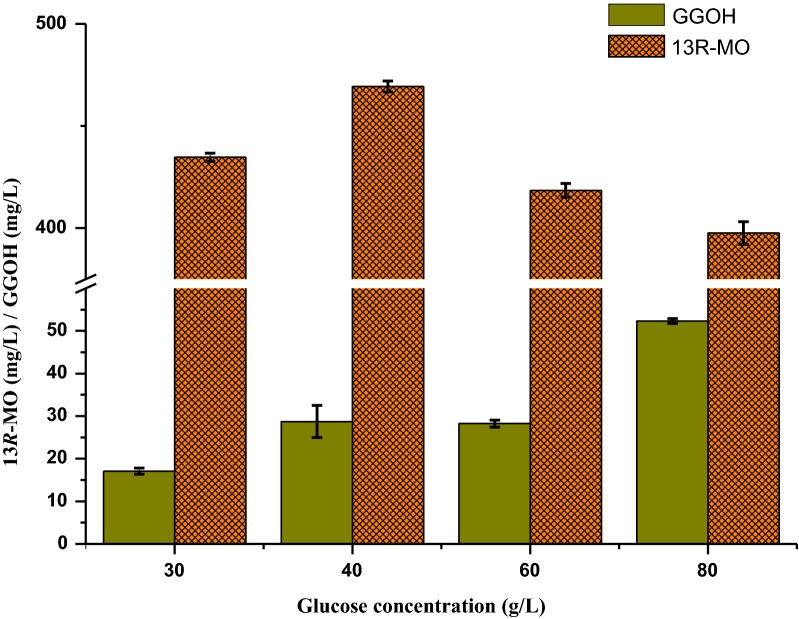
13*R*-MO and GGOH production by the strain LZJ7 in shake flasks with various glucose concentrations. YPD media containing 30 g/L, 40 g/L, 60 g/L, and 80 g/L of glucose were used for fermentation. Three independent experiments were performed, and the error bars represent their standard deviation

The strain LZJ7 was selected for the 5 L bioreactor scale-up 13*R*-MO production. The glucose substrate concentration was optimized in shake flasks; as shown in Fig. 4. The highest 13*R*-MO titer, 469.26 mg/L, was achieved with a glucose concentration of 40 g/L, therefore, 40 g/L of glucose was used in the 5 L bioreactor scale-up fermentation.

**Fig. 5:**
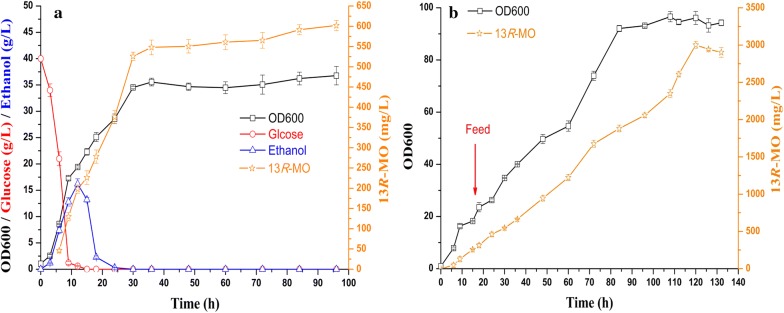
Production of 13*R*-MO by batch and fed-batch cultivation in a 5 L bioreactor. **a** Batch fermentation using LZJ7 in the 5 L bioreactor. YPD medium containing 40 g/L of glucose was used for the fermentation; fermentation was conducted at 30 °C with an airflow rate of 2 vvm, and the pH was automatically maintained at 5.5. **b** Fed-batch fermentation using LZJ7 in the 5 L bioreactor. The feed solution was added at 18 h, as indicated by the red arrow

The results of the batch and fed-batch fermentation of LZJ7 in the 5 L bioreactor are shown in Fig. 5. Glucose was quickly exhausted within 20 h, while the ethanol concentration reached 16.2 g/L at 12 h (Fig. 5a). With the depletion of ethanol (about 30 h), the 13*R*-MO titer increased to 602.05 mg/L, which was 1.28-fold higher than that in the shake flasks. According to the substrate consumption curve, the feeding medium was added at 18 h and 3 g/L of 13*R*-MO was obtained at 120 h (Fig. 5b).

## Discussion

To date, 15,804 diterpenoid compounds have been discovered in plants [28]. However, most diterpenoid compounds cannot be produced commercially due to their low concentrations in the native host. An efficient diterpenoid-producing yeast cell factory would be of broad interest as it could be used to produce a variety of high-value diterpenoid compounds in a cost-effective manner. In yeast, terpenoid synthesis begins with the common precursors IPP and DMAPP, which are derived from the MVA pathway in the cytoplasm. The diterpenoid precursor GGPP is systematically synthesized by Erg20p and Bts1p in *S. cerevisiae*. Erg9p plays a vital role in the distribution of FPP flux between FPP-derived terpenoid products and sterols [29]. In our previous study, the *ERG9* promoter was replaced with *MET3p* in *S. cerevisiae* to produce levopimaradiene, a type of diterpene produced by plants at a titer 8.5-fold higher than that produced by its parental strain. However, this strategy needs the addition of methionine, which could be metabolized by the cell [18, 29]. In this study, a well-established sesquiterpene-producing yeast platform, LW03, was used to produce 13*R*-MO by overexpressing *CfTPS2* and *CfTPS3*, but the titer of 13*R*-MO did not increase significantly. In our previous study, LW03 was found to accumulate squalene (3.73 mg/g CDW) during fermentation anaphase, although *ERG9* was controlled by *HXT1p*, an efficient promoter for repressing *ERG9* transcription under glucose-limiting conditions [29]. To block squalene synthesis, a PEST (rich in Pro, Glu/Asp, Ser, and Thr) sequence was added to the C-terminal of Erg9p (LZJ2) to trigger endoplasmic reticulum-associated protein degradation [27]. This strategy improved the titer of the sesquiterpene nerolidol by 86% and decreased the level of squalene to that of the wild-type control strain [27]. Indeed, the squalene content of the LZJ2 strain was decreased to 0.12 mg/g CDW, but the 13*R*-MO titer remained low and the FOH and GGOH titers increased by 3.53-fold and 1.87-fold, respectively. The level of FOH and GGOH accumulation often reflects the intracellular content of the FPP and GGPP pools [3]. The increases observed in the FPP and GGPP pools indicate that combining the regulation of transcription and protein destabilization effectively reduces FPP consumption by Erg9p and redirects the carbon flux from squalene to FPP and GGPP.

In *S. cerevisiae*, the diterpenoid precursor GGPP is synthesized by the condensation of FPP and IPP by Bts1p. The coupling of FPP and GGPP synthesis was found to be an important limitation of diterpene production [20]. Selection of GGPP synthases from different sources and overexpression of native *BTS1* were all attempted to improve the supply of GGPP [21]. An Erg20p mutant (Erg20^F96C^p) was constructed to produce GGPP, and was found to efficiently produce diterpenes and carotenoids [20]. To draw the FPP pool to GGPP, *BTS1* and *ERG20*^*F96C*^ were co-expressed in LZJ3 causing an increase in FOH, GGOH, and 13*R*-MO production compared with the parental strain LZJ2. Although Erg20^F96C^p produced GGPP, no obvious effect on FPP formation was observed in vitro [20]. To further combine FPP and GGPP synthesis, Bts1p was fused with Erg20p or Erg20^F96C^p; the two fusion proteins were able to effectively draw FPP to GGPP, resulting in a decrease in FOH accumulation of approximately 50% compared with the parental strain LZJ3. The Bts1-GGGS-Erg20^F96C^p fusion protein was found to be more effective for GGPP production and led to about threefold GGOH production when compared with Bts1p and Erg20^F96C^p co-expression. The 13*R*-MO titer was also increased to 23.21 mg/L. Erg20p and Bts1p fusion is an efficient method of improving diterpene production [30, 31], and in our study the fusion of mutant Erg20p (Erg20^F96C^p) and Bts1p enabled FPP and GGPP to be coupled more efficiently.

In plants, GGPP is synthesized in plastids through the methylerythritol 4-phosphate pathway [32]. A plastid transit peptide located at the N-terminal of diterpene synthase was found to influence its catalytic activity when the enzyme was expressed in *S. cerevisiae* [23]. In this study, the N-terminal plastid transit peptides of CfTPS2 and CfTPS3 were truncated according to the predictions of the ChloroP software, and the resulting enzymes were integrated into the *δ* sites of LZJ6 [12]. 13*R*-MO production increased by 7.6-fold (176.8 mg/L) compared with the level produced by the parental strain LZJ5. The accumulation of GGOH and FOH decreased significantly, which indicated that the precursor pool was successfully converted to 13*R*-MO. The two limiting-nodes, *tHMG1* and *BTS1-GGGS-ERG20*^*F96C*^, were further over-expressed in LZJ7, resulting in a higher level of 13*R*-MO production (328.15 mg/L).

## Conclusions

An efficient 13*R*-MO yeast cell factory was constructed in this study. Stepwise metabolic engineering strategies were used to modify the MVA pathway leading to 13*R*-MO production. The highly efficient production of GGPP from FPP and the high activity of diterpene synthase were the two limiting steps for diterpenoids production. Transcription and protein level regulation were combined to transfer the metabolic flux from squalene to FPP and then to GGPP, which was an efficient way of modifying the Erg9p node. This strategy may also work well for sesquiterpene production in yeast. In summary, the engineered *S. cerevisiae* strain LZJ7 produced 602.05 mg/L of the diterpene 13*R*-MO in batch culture with 40 g/L of glucose, and the titer reached 3 g/L with fed-batch fermentation. This study provides a new strategy for the highly efficient production of diterpenoids and potentially even tetraterpenes by using the microbial cell factory.

## Methods

### Strains, media, and culture conditions

*Saccharomyces cerevisiae* W303-1a (*MATa*; *leu2-3112*; *trp1-1*; *can1-100*; *ura3-1*; *ade2-1*; *his3-11,15*) was used as the original strain and was maintained in our laboratory. YPD medium (2% glucose, 1% yeast extract, and 2% peptone) was used for yeast cultivation, and SC medium (2% glucose, 0.67% YNB, and amino acid drop-out mix) was used for recombinant yeast strains selection. For shake flask cultivation, a single yeast colony was inoculated in 3 mL of YPD medium and cultivated overnight; the overnight culture was transferred to 250 mL shake flasks containing 30 mL of YPD medium with an initial OD600 of 0.05 and incubated for 96 h at 30 °C, 220 rpm. Dodecane (10%) was added for biphasic fermentation at 6 h after inoculation. YPD medium with different glucose concentrations (30 g/L, 40 g/L, 60 g/L, and 80 g/L) was used to optimize the fermentation of the strain LZJ7 in shake flasks. All fermentations were performed in triplicate.

### Engineering of *S. cerevisiae* strain for 13*R*-MO production

The strains established in this study are listed in Table 2. The primers used for strain engineering in this study are listed in Additional file 1: Table S1. *CfTPS2* (GenBank: KF444507) and *CfTPS3* (GenBank: KF444508) were synthesized and codon-optimized for *S. cerevisiae* by Jinsirui Biotechnology Co., Ltd. (Nanjing, China), and the sequences are listed in Additional file 1. The gene expression modules were constructed according to our previous methods (Additional file 1: Figure S1). Briefly, the promoters, MVA pathway genes, and terminators were amplified from *S. cerevisiae* W303-1a genomic DNA, and the fragments were purified for gene expression module construction via fusion PCR. The Bts1-Erg20p fusion protein was constructed with a widely used linker, “Gly-Gly-Gly-Ser”. The primer pairs ERG20-F/F96C-R and F96C-F/ERG20-R were used to construct Erg20p^F96C^. The constructed gene expression modules, homologous arms of the integration sites and selection markers were transformed into *S. cerevisiae*, according to the methods of Zhao et al. [33].

**Table 2:** Strains used in this study

Strain	Description	Source
W303-1a	*MATa*; *leu2-3112*; *trp1-1*; *can1-100*; *ura3-1*; *ade2-1*; *his3-11,15*	Our lab
GW-1	W303-1a, *ura3*:: *P*_*PGK1*_*-CfTPS2-T*_*ADH1*_*, P*_*TDH3*_*-CfTPS3-T*_*TDH2*_	This study
LZJ1	*ade2*:: *P*_*PGK1*_*- tHMG1-T*_*PGK1*_*, T*_*TDH3*_*-ERG20-T*_*ERG20*_; *P*_*ERG9*_*::P*_*HXT1*_*-ERG9*; *ura3*:: *P*_*PGK1*_*-CfTPS2-T*_*ADH1*_*, P*_*TDH3*_*-CfTPS3-T*_*TDH2*_	[18]
LZJ2	*ade2*:: *P*_*PGK1*_*- tHMG1-T*_*PGK1*_*, T*_*TDH3*_*-ERG20-T*_*ERG20*_; *P*_*ERG9*_*::P*_*HXT1*_*-ERG9-PEST*; *ura3*:: *P*_*PGK1*_*-CfTPS2-T*_*ADH1*_*, P*_*TDH3*_*-CfTPS3-T*_*TDH2*_	This study
LZJ3	*ade2*:: *P*_*PGK1*_*- tHMG1-T*_*PGK1*_*, T*_*TDH3*_*-ERG20-T*_*ERG20*_; *P*_*ERG9*_::*P*_*HXT1*_*-ERG9-PEST*; *ura3*:: *P*_*PGK1*_*-CfTPS2-T*_*ADH1*_*, P*_*TDH3*_*-CfTPS3-T*_*TDH2*_; HO::* P*_*TDH3*_*-BTS1-T*_*CYC1*_,* P*_*PGK1*_*-ERG20*^F96C^*-T*_*ERG20*_	This study
LZJ4	*ade2*:: *P*_*PGK1*_*- tHMG1-T*_*PGK1*_*, T*_*TDH3*_*-ERG20-T*_*ERG20*_; *P*_*ERG9*_::*P*_*HXT1*_*-ERG9-PEST*; *ura3*:: *P*_*PGK1*_*-CfTPS2-T*_*ADH1*_*, P*_*TDH3*_*-CfTPS3-T*_*TDH2*_; HO::* P*_*TDH3*_*-BTS1-GGGS-ERG20-T*_*ERG20*_	This study
LZJ5	*ade2*:: *P*_*PGK1*_*- tHMG1-T*_*PGK1*_*, T*_*TDH3*_*-ERG20-T*_*ERG20*_; *P*_*ERG9*_::*P*_*HXT1*_*-ERG9-PEST*; *ura3*:: *P*_*PGK1*_*-CfTPS2-T*_*ADH1*_*, P*_*TDH3*_*-CfTPS3-T*_*TDH2*_; HO::* P*_*TDH3*_*-BTS1-GGGS-ERG20*^F96C^*-T*_*ERG20*_	This study
LZJ6	*ade2*:: *P*_*PGK1*_*- tHMG1-T*_*PGK1*_*, T*_*TDH3*_*-ERG20-T*_*ERG20*_; *P*_*ERG9*_::*P*_*HXT1*_*-ERG9-PEST*; *ura3*:: *P*_*PGK1*_*-CfTPS2-T*_*ADH1*_*, P*_*TDH3*_*-CfTPS3-T*_*TDH2*_; HO::* P*_*TDH3*_*-BTS1-GGGS-ERG20*^F96C^*-T*_*ERG20*_; *δ:: P*_*TEF1*_*-tCfTPS2-T*_*ADH1*_*, P*_*TDH3*_*-tCfTPS3-T*_*TDH2*_	This study
LZJ7	*ade2*:: *P*_*PGK1*_*- tHMG1-T*_*PGK1*_*, T*_*TDH3*_*-ERG20-T*_*ERG20*_; *P*_*ERG9*_::*P*_*HXT1*_*-ERG9-PEST*; *ura3*:: *P*_*PGK1*_*-CfTPS2-T*_*ADH1*_*, P*_*TDH3*_*-CfTPS3-T*_*TDH2*_; HO::* P*_*TDH3*_*-BTS1-GGGS-ERG20*^F96C^*-T*_*ERG20*_; *δ:: P*_*PGK1*_*-tCfTPS2-T*_*ADH1*_*, P*_*TDH3*_*-tCfTPS3-T*_*TDH2*_; rDNA:: *P*_*PGK1*_*- tHMG1-T*_*PGK1*_*, P*_*TDH3*_*-BTS1-GGGS-ERG20*^F96C^*-T*_*ERG20*_	This study

### Detection and quantification of 13*R*-MO

The yeast cells were harvested by centrifugation, then the cell pellets and supernatants were extracted with hexane. The hexane phase was filtrated and analyzed using GC or GC–MS. For biphasic-fermentation, the dodecane layer was separated by centrifugation (12,000 rpm, 10 min) and subsequently analyzed with GC or GC–MS. The samples (1 μL) were analyzed using a Shimadzu GCMS-TQ8030 instrument equipped with an HP-5ms GC column (Agilent technologies, 30 m × 0.250 mm × 0.25 μm) and with helium as the carrier gas. The following temperature gradient program was used: injection temperature, 250 °C; 60 °C for 1 min, 15 °C/min to 200 °C, 10 °C/min to 280 °C, and hold for 2 min; 20 °C/min to 300 °C; and hold for 2 min. The ion source temperature was set to 300 °C, and the spectra were scanned from 30 to 550 m/z. 1-Eicosene was used as the internal standard for 13*R*-MO quantification, according to the method reported by Elias et al. [26].

### Metabolite extraction and analysis

Glucose was quantified with an SBA-40C bio-analyzer (Shandong Academy of Sciences, China), according to the manufacturer’s instructions. The yeast cells were collected by centrifugation, washed three times and OD600 was measured using a spectrophotometer (Oppler, 752N, China). Squalene was extracted and quantified using HPLC (Elite P230II high-pressure pump system coupled with UV detection at 203 nm; Hypersil C18 column, 4.6 mm × 250 mm, 5 μm; Elite Analytical Instruments Co., Ltd., Dalian, China), according to our previously described methods [33]. FOH in the dodecane layer was quantified using a standard curve and GC. The squalene and FOH standards were purchased from Sigma (USA).

### Batch and fed-batch scale-up fermentation in a 5 L bioreactor

For batch and fed-batch fermentation, OYPD medium (4% glucose, 1% yeast extract, and 2% peptone) was used as the seed and fermentation medium. The strain LZJ7 was prepared in a 500 mL shake-flask containing 100 mL of OYPD medium at 30 °C and 220 rpm for 16 h. The pre-culture was then inoculated at 10 vol % in a 5 L bioreactor (ShangHai BaiLun Bio) containing 3 L (batch) or 2 L (fed-batch) of OYPD medium. The pH was automatically maintained at 5.5 using 5 N H_2_SO_4_ and NH_3_·H_2_O. The temperature was maintained at 30 °C, and the air flow rate was set at 2 vvm. The dissolved oxygen (DO) content was maintained above 35% by adjusting the agitation speed between 300 and 600 rpm. For two-phase fermentation, 10 vol % of dodecane was added after 6 h of cultivation.

The feed solution was prepared according to our previously reported methods and was added automatically according to the pH and DO after 18 h [18]. When the pH was above 5.5 and DO was above 35%, the feed solution was added at a rate of 2 mL/min. NH_3_·H_2_O was used as the nitrogenous source and was added automatically when the pH dropped below 5.5.

## Additional file

**Additional file 1: Table S1.** Primers used in this study. **Figure S1.** Expression cassettes construction and insertion. **Table S2.** Synthesized DNA sequences.

## Abbreviations

13*R*-MO: 13*R*-manoyl oxide
GGPP: geranylgeranyl diphosphate
MVA: mevalonate
IPP: isopentenyl diphosphate
DMAPP: dimethylallyl diphosphate
GPP: geranyl diphosphate
FPP: farnesyl diphosphate
Acetyl-CoA: acetyl coenzyme A
HMG-CoA: 3-hydroxy-3-methylglutaryl-CoA
*tHMG*1: truncated HMG-CoA reductase gene
FOH: farnesol
*ERG9*: squalene synthetase gene
GGOH: geranylgeraniol
