# Supplementary material for: High-titer production of 13R-manoyl oxide in metabolically engineered Saccharomyces cerevisiae
Source: Microb Cell Fact. 2019 Apr 24;18:73. doi: 10.1186/s12934-019-1123-z (PMC6480505; doi:10.1186/s12934-019-1123-z)
Supplement: Supplementary file 1 — Additional file 1: Table S1. Primers used in this study. Figure S1. Expression cassettes construction and insertion. Table S2. Synthesized DNA sequences. [file 12934_2019_1123_MOESM1_ESM.docx]

Additional file

**High-titer production of 13*R*-manoyl oxide in metabolically engineered *Saccharomyces cerevisiae***

Chuanbo Zhang^a^, Haiyan Ju^a^, Chun-Zhe Lu^a^, Fanglong Zhao^a^, Jingjing Liu^a^, Xiaoyan Guo^a^, Yufen Wu^a^, Guang-Rong Zhao^a,b,c^, Wenyu Lu^a,b,c,^*

^a^ School of Chemical Engineering and Technology, Tianjin University, Tianjin 300350, P. R. China

^b^ Key Laboratory of System Bioengineering (Tianjin University), Ministry of Education, Tianjin 300350, P. R. China

^c^ SynBio Research Platform, Collaborative Innovation Center of Chemical Science and Engineering (Tianjin), Tianjin 300350, P. R. China

* Correspondence to W. Y. Lu, School of Chemical Engineering and Technology, Tianjin University, Tianjin 300350, PR China. Tel: +86-22-85356523, Fax: +86-22-27400973. E-mail address: [wenyulu@tju.edu.cn](mailto:wenyulu@tju.edu.cn)

**Table S1. Primers used in this study**

| **Name** | | **Primer sequence (5’-3’)** | |
| --- | --- | --- | --- |
| ***TPS2* and *TPS3* gene expression cassettes construction** | | | |
| URA3U-F | | GACGTTGAAATTGAGGCTACTGCG | |
| PGK1-URA3U-R | | ATAATATCTGTGCGTGACCTAATGCTTCAACTAAC | |
| URA3U- PGK1-F | | GTTGAAGCATTAGGTCACGCACAGATATTATAACATC | |
| TPS2- PGK1-R | | CATCAACATCTTCATTGTTTTATATTTGTTGTAAAAAGTAG | |
| PGK1-TPS2-F | | ACAAATATAAAACAATGAAGATGTTGATGATCAAGTC | |
| ADH1-TPS2-R | | ATAAGAAATTCGCTTAAACAACTGGTTCAAACAAAAC | |
| TPS2-ADH1-F | | TGAACCAGTTGTTTAAGCGAATTTCTTATGATTTATG | |
| TDH3-ADH1-R | | CTAACATTCAACGCTAGTATAGATCATGATACATAAAAGC | |
| ADH1-TDH3-F | | GCTTTTATGTATCATGATCTATACTAGCGTTGAATGTTAG | |
| TPS3-TDH3-R | | ATGAAGACATTTTGTTTGTTTATGTGTGTTTATTC | |
| TDH3-TPS3-F | | CACATAAACAAACAAAATGTCTTCATTGGCTGGTAATTTG | |
| TDH2-TPS3-R | | AAGGAGTTAAATTTAATTTGAAACACAAGACATC | |
| TPS3-TDH2-F | | GTGTTTCAAATTAAATTTAACTCCTTAAGTTACTTTAATG | |
| HIS3-TDH2-R | | CAGGCATGCAAGCGCGAAAAGCCAATTAGTG | |
| TDH2-HIS3-F | | CACTAATTGGCTTTTCGCGCTTGCATGCCTG | |
| URA3D-HIS3-R | | GTAGAGACCACATCTAGCACGTGATGAATTCGAG | |
| HIS3-URA3D-F | | ATTCATCACGTGCTAGATGTGGTCTCTACAGG | |
| URA3D-R | | CAAGCCTTGTCCCAAGGCAGCG | |
| ***ERG9-PEST* gene expression cassettes construction** | | | |
| ERG9C-F | TGGTCGATGGTAGATCCTTCTGGC | | |
| PEST-ERG9-R | GTTCAAGTTGGATGCCGCTCTGTGTAAAGTGTATAT | | |
| ERG9-PEST-F | CTTTACACAGAGCGGCATCCAACTTGAACATTTCG | | |
| CYC1-PEST-R | CTAATTACATGACTATATTACTTGGGTATTGCCCATAC | | |
| PEST-CYC1-F | ACCCAAGTAATATAGTCATGTAATTAGTTATGTCACGCT | | |
| HIS3-CYC1-R | TAGAGTCGACCAAATTAAAGCCTTCGAGC | | |
| CYC1-HIS3-F | AAGGCTTTAATTTGGTCGACTCTAGAGGATC | | |
| Terg9-HIS3-R | ATTTGGCGCAGACTATAGCACGTGATGAATTCGAG | | |
| HIS3-Terg9-F | TCATCACGTGCTATAGTCTGCGCCAAATAACATAAAC | | |
| Terg9-R | TATACATGTCAACGTTAGCATCTTGAAT | | |
| **ERG20 mutation** | | | |
| ERG20-F | CAATAAACTCAAAAATATTAC | | |
| F96C-R | TCGGCGACCAAGCAGTAAGCCTGCAACAACTC | | |
| F96C-F | CAGGCTTACTGCTTGGTCGCCGAT | | |
| ERG20-R | TCTAGAGTCGACCTTGGACTAGTCACGTGGAAC | | |
| ***BTS1* and *ERG20/ERG20^F96C^* gene expression cassettes construction** | | | |
| HOU-F | | TCCCAGGCGTAGAACAGTTTATCAG | |
| TDH3-HOU-R | | TCAACGCTAGTATGATCCAAGCTATCTACTGAG | |
| HOU-TDH3-F | | AGATAGCTTGGATCATACTAGCGTTGAATGTTAG | |
| BTS1-TDH3-R | | ATCTTGGCCTCCATTTTGTTTGTTTATGTGTGTTTATTC | |
| TDH3-BTS1-F | | CATAAACAAACAAAATGGAGGCCAAGATAGATGAG | |
| CYC1-BTS1-R | | GTGACATAACTAATTCACAATTCGGATAAGTGGTCT | |
| BTS1-TCYC1-F | | TATCCGAATTGTGAATTAGTTATGTCACGCTTAC | |
| PGK1-TCYC1-R | | CAAATGCCTAATATTCAAATTAAAGCCTTCGAGCGTC | |
| TCYC1-PGK1-F | | CTCGAAGGCTTTAATTTGAATATTAGGCATTTGCAAGAATTACT | |
| ERG20-PGK1-R | | TCTGAAGCCATACTAGTATATTTGTTGTAAAAAG | |
| PGK1-ERG20-F | | GTAATTATCTACTTTTTACAACAAATATACTAGTATGGCTTCAGAAAAAGAAATTAG | |
| TRP1-TERG20-R | | GGCATGCAAGCTTGCAATGTTCGAGAGATGAGGTC | |
| TERG20-TRP1-F | | TCTCGAACATTGCAAGCTTGCATGCCTGC | |
| HOD-TRP1-R | | GACATACCAAGATAGCACGTGATGAATTCGAG | |
| TRP1-HOD-F | | CATCACGTGCTATCTTGGTATGTCAGCTACT | |
| HOD-R | | CACTTCACGTGCTTCTGGTACATACTTG | |
| ***BTS1-GGGS-ERG20^F96C^*** **gene expression cassette construction** | | | |
| Lingker-BTS1-R | | | TGAAGCCATAGAACCACCACCCAATTCGGATAAGTGGTCTAT |
| Lingker-ERG20-F | | | GGTGGTGGTTCTATGGCTTCAGAAAAAGAAATT |
| ***TPS2* and *TPS3* truncation** | | | |
| TEF1-tTPS2-F | | | AGTTTTAATTACAAAATGGTTGCATCTTTAGATGCTTTG |
| TDH3-tTPS3-F | | | CATAAACAAACAAAATGTCAGCTGCAGTTAAATGTTCATTGACT |
| ***P_PGK1_-tTPS2-T_ADH1_, P_TDH3_-tTPS3-T_TDH2_*** **gene expression cassettes inserted in** δ **sites** | | | |
| δ2-F | | | GCTTCGGTTACTTCTAAGGAAGTCCACAC |
| TEF1-δ2-R | | | GTGGGGGATCACTTTGGAAAGTCATTAGGTGAG |
| δ2 -TEF1-F | | | TAATGACTTTCCAAAGTGATCCCCCACACACCAT |
| tTPS2- TEF1-R | | | ATCTAAAGATGCAACCATTTTGTAATTAAAACTTAGATTAG |
| TEF1-tTPS2-F | | | AGTTTTAATTACAAAATGGTTGCATCTTTAGATGCTTTG |
| ADH1-tTPS2-R | | | ATAAGAAATTCGCTTAAACAACTGGTTCAAACAAAAC |
| tTPS2-ADH1-F | | | TGAACCAGTTGTTTAAGCGAATTTCTTATGATTTATG |
| TDH3-ADH1-R | | | CTAACATTCAACGCTAGTATAGATCATGATACATAAAAGC |
| ADH1-TDH3-F | | | GCTTTTATGTATCATGATCTATACTAGCGTTGAATGTTAG |
| tTPS3-TDH3-R | | | ATTTAACTGCAGCTGACATTTTGTTTGTTTATGTGTG |
| TDH3-tTPS3-F | | | CATAAACAAACAAAATGTCAGCTGCAGTTAAATGTTCATTGACT |
| TDH2-tTPS3-R | | | AAGGAGTTAAATTTAATTTGAAACACAAGACATC |
| tTPS3-TDH2-F | | | GTGTTTCAAATTAAATTTAACTCCTTAAGTTACTTTAATG |
| LEU2-TDH2-R | | | CAGGCATGCAAGCGCGAAAAGCCAATTAGTG |
| TDH2-LEU2-F | | | CACTAATTGGCTTTTCGCGCTTGCATGCCTG |
| δ1-LEU2-R | | | GTTGATTTCTATTCCAACACCCGGGATAACTTCGTATAATG |
| LEU2-δ1-F | | | CATTATACGAAGTTATCCCGGGTGTTGGAATAGAAATCAAC |
| δ1-R | | | CACAGGCGCTACCATGAGAATTG |
| ***tHMG1 and BTS1-GGGS-ERG20*^F96C^ gene expression cassettes inserted in *rDNA* sites** | | | |
| rDNA2-F | | CCGGGGCACCTGTCACTTTGGAAAAAAAATATACGCTAAGA | |
| PGK1-rDNA2-R | | TGGTGGAACCTGATTAGAGGAAATATTTTAGATTCCTGACTTCAACTC | |
| rDNA2-PGK1-F | | GAGTTGAAGTCAGGAATCTAAAATATTTCCTCTAATCAGGTTCCAC | |
| tHMG1-PGK1-R | | ATTGGTTAAAACTGGCATTGTTTTATATTTGTTGTAAAAAGTAG | |
| PGK1-tHMG1-F | | CAACAAATATAAAACAATGCCAGTTTTAACCAATAAAACAGTC | |
| PGK1T-tHMG1-R | | CTATCGATTTCAATTCAATTCAATTTAGGATTTAATGCAGGTGAC | |
| tHMG1-PGK1T-F | | GTCACCTGCATTAAATCCTAAATTGAATTGAATTGAAATCGATAG | |
| TDH3-PGK1T-R | | TAACATTCAACGCTAGTATCACTATACTGGATCTAAAGAGTACAATAG | |
| PGK1T-TDH3-F | | CTCTTTAGATCCAGTATAGTGATACTAGCGTTGAATGTTAGCG | |
| BTS1-TDH3-R | | ATCTTGGCCTCCATTTTGTTTGTTTATGTGTGTTTATTC | |
| TDH3-BTS1-F | | CATAAACAAACAAAATGGAGGCCAAGATAGATGAG | |
| URA3-TERG20-R | | GGCATGCAAGCTTGCAATGTTCGAGAGATGAGGTC | |
| TERG20-URA3-F | | TCTCGAACATTGCAAGCTTGCATGCCTGC | |
| rDNA1-URA3-R | | TGCTACTCTCATACGTGATGAATTCGAGCTC | |
| URA3-rDNA1-F | | GAATTCATCACGTATGAGAGTAGCAAACGTAAG | |
| rDNA1-R | | GCGGAAAATACGGAAACGCGCGGGAACATACAA | |

**
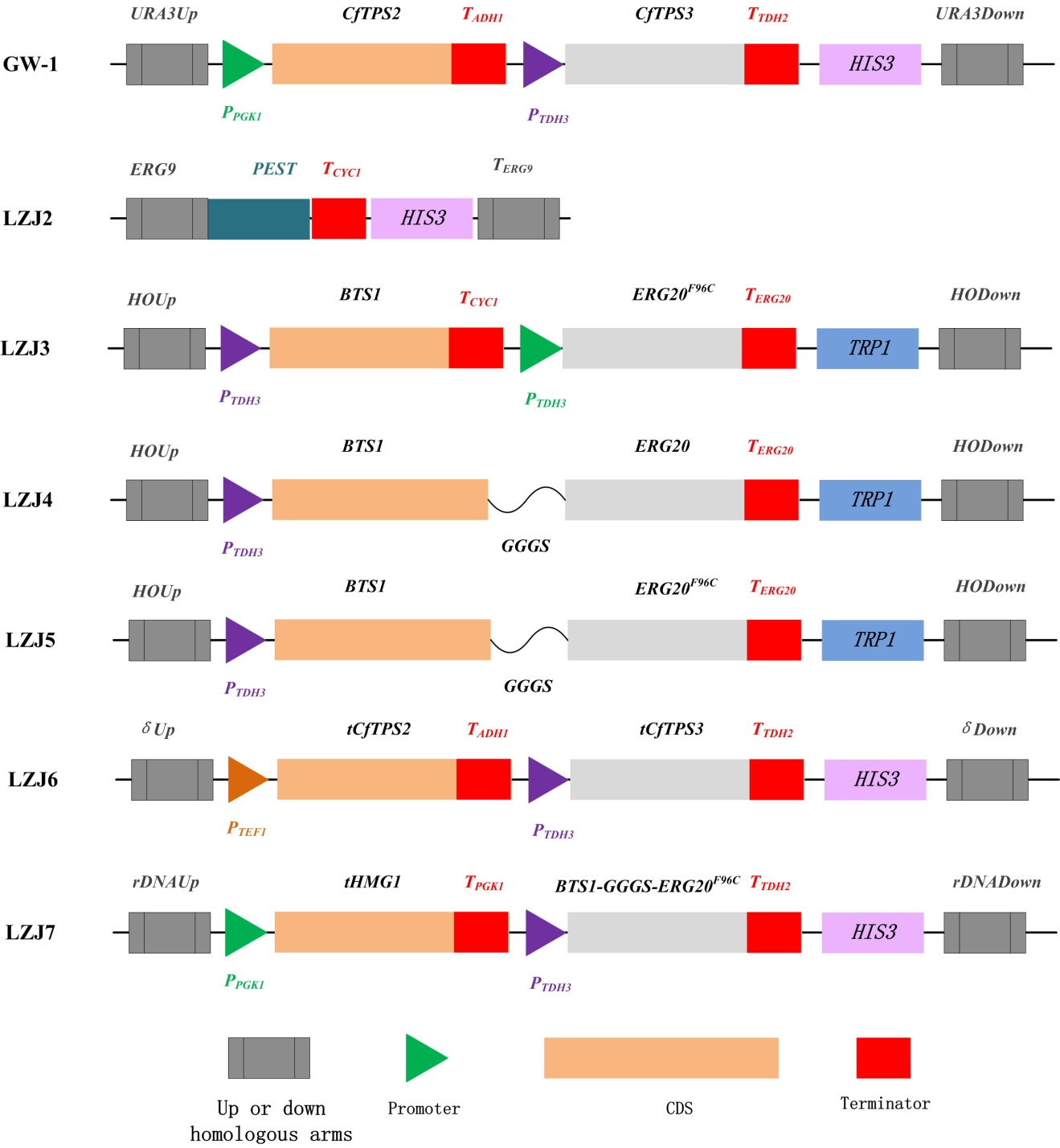
**

**Figure S1.** **Expression cassettes construction and insertion**

**Table S2.** Synthesized DNA sequences

| **Name** | sequences |
| --- | --- |
| CfTPS2 | ATGAAGATGTTGATGATCAAGTCACAATTCAGAGTTCATTCTATTGTTTCAGCATGGGCTAACAACTCAAATAAGAGACAATCTTTGGGTCATCAAATCAGAAGAAAGCAAAGATCACAAGTTACAGAATGTAGAGTTGCATCTTTAGATGCTTTGAACGGTATTCAAAAGGTTGGTCCAGCTACTATTGGTACACCAGAAGAAGAAAATAAGAAAATTGAAGATTCTATCGAATATGTTAAAGAATTGTTAAAAACAATGGGTGACGGTAGAATTTCTGTTTCACCATACGATACTGCAATCGTTGCTTTGATTAAAGATTTGGAAGGTGGTGACGGTCCAGAATTTCCATCATGTTTGGAATGGATTGCACAAAATCAATTAGCTGATGGTTCTTGGGGTGACCATTTCTTTTGTATCTATGATAGAGTTGTTAATACAGCTGCATGTGTTGTTGCATTGAAGTCTTGGAACGTTCATGCAGATAAGATCGAAAAGGGTGCTGTTTACTTGAAGGAAAACGTTCATAAGTTGAAAGATGGTAAAATTGAACATATGCCAGCAGGTTTTGAATTTGTTGTTCCAGCTACTTTAGAAAGAGCAAAAGCTTTGGGTATTAAAGGTTTGCCATACGATGATCCTTTTATTAGAGAAATCTATTCAGCTAAGCAAACTAGATTGACAAAGATCCCAAAGGGTATGATCTATGAATCTCCAACATCATTGTTGTACTCTTTAGATGGTTTGGAAGGTTTAGAATGGGATAAGATCTTGAAGTTGCAATCTGCAGATGGTTCTTTTATTACTTCTGTTTCTTCAACAGCTTTCGTTTTTATGCATACTAACGATTTGAAATGTCATGCTTTTATTAAAAATGCTTTGACAAACTGTAATGGTGGTGTTCCACATACTTACCCAGTTGATATCTTCGCAAGATTATGGGCTGTTGATAGATTGCAAAGATTGGGTATTTCAAGATTTTTCGAACCAGAAATTAAATACTTGATGGATCATATCAACAACGTTTGGAGAGAAAAGGGTGTTTTCTCTTCAAGACATTCACAATTCGCTGATATCGATGATACATCTATGGGTATTAGATTGTTGAAGATGCATGGTTACAATGTTAATCCAAACGCATTGGAACATTTCAAGCAAAAGGATGGTAAATTCACTTGTTACGCTGATCAACATATCGAATCTCCATCACCAATGTACAATTTGTACAGAGCTGCACAATTGAGATTCCCAGGTGAAGAAATCTTGCAACAAGCATTGCAATTCGCTTACAATTTCTTGCATGAAAATTTGGCTTCAAACCATTTCCAAGAAAAGTGGGTTATTTCTGATCATTTGATCGATGAAGTTAGAATCGGTTTGAAGATGCCATGGTATGCAACTTTGCCAAGAGTTGAAGCTTCATACTACTTACAACATTACGGTGGTTCTTCAGATGTTTGGATCGGTAAAACATTGTACAGAATGCCAGAAATCTCTAACGATACTTACAAGATCTTGGCACAATTGGATTTCAATAAGTGTCAAGCTCAACATCAATTAGAATGGATGTCAATGAAGGAATGGTATCAATCTAACAACGTTAAGGAATTTGGTATCTCTAAGAAAGAATTGTTGTTGGCATACTTTTTGGCTGCAGCTACTATGTTTGAACCAGAAAGAACACAAGAAAGAATTATGTGGGCTAAGACTCAAGTTGTTTCAAGAATGATCACATCATTTTTGAATAAGGAAAATACTATGTCATTTGATTTGAAAATTGCATTGTTGACACAACCACAACATCAAATTAATGGTTCTGAAATGAAGAATGGTTTGGCTCAAACTTTACCAGCAGCTTTTAGACAATTGTTGAAGGAATTTGATAAGTACACTAGACATCAATTGAGAAACACATGGAATAAGTGGTTGATGAAGTTGAAGCAAGGTGACGATAACGGTGGTGCAGATGCTGAATTATTGGCAAACACATTGAACATCTGTGCTGGTCATAACGAAGATATCTTGTCTCATTACGAATACACTGCCTTGTCTTCATTGACAAATAAGATCTGTCAAAGATTATCACAAATTCAAGATAAGAAAATGTTGGAAATCGAAGAAGGTTCTATTAAAGATAAGGAAATGGAATTGGAAATTCAAACATTAGTTAAATTAGTTTTGCAAGAAACTTCAGGTGGTATCGATAGAAACATCAAGCAAACATTTTTGTCTGTTTTTAAAACATTCTACTACAGAGCATACCATGATGCTAAGACTATCGATGCTCATATCTTCCAAGTTTTGTTTGAACCAGTTGTTTAA |
| CfTPS3 | ATGTCTTCATTGGCTGGTAATTTGAGAGTTATTCCATTTTCTGGTAACAGAGTTCAAACTAGAACAGGTATTTTGCCAGTTCATCAAACTCCAATGATCACATCTAAATCTTCAGCTGCAGTTAAATGTTCATTGACTACACCAACTGATTTGATGGGTAAAATTAAAGAAGTTTTTAATAGAGAAGTTGATACTTCTCCAGCTGCAATGACTACACATTCTACAGATATCCCATCAAATTTGTGTATCATCGATACTTTGCAAAGATTGGGTATCGATCAATACTTCCAATCTGAAATCGATGCTGTTTTGCATGATACATACAGATTGTGGCAATTGAAAAAGAAAGATATTTTCTCTGATATCACTACACATGCTATGGCTTTTAGATTGTTGAGAGTTAAGGGTTACGAAGTTGCATCAGATGAATTGGCTCCATACGCAGATCAAGAAAGAATTAATTTGCAAACTATTGATGTTCCAACAGTTGTTGAATTGTATAGAGCTGCACAAGAAAGATTGACTGAAGAAGATTCTACATTGGAAAAGTTGTACGTTTGGACTTCAGCATTTTTGAAGCAACAATTGTTGACAGATGCAATCCCAGATAAGAAATTGCATAAGCAAGTTGAATACTACTTGAAAAATTATCATGGTATTTTAGATAGAATGGGTGTTAGAAGAAATTTGGATTTGTACGATATCTCTCATTACAAATCATTGAAGGCTGCACATAGATTCTACAATTTGTCTAACGAAGATATCTTGGCTTTCGCAAGACAAGATTTCAACATCTCACAAGCTCAACATCAAAAGGAATTGCAACAATTGCAAAGATGGTATGCAGATTGTAGATTGGATACTTTGAAGTTCGGTAGAGATGTTGTTAGAATCGGTAATTTCTTGACATCTGCTATGATTGGTGACCCAGAATTATCAGATTTGAGATTGGCTTTCGCAAAGCATATCGTTTTGGTTACTAGAATCGATGATTTCTTTGATCATGGTGGTCCAAAGGAAGAATCTTACGAAATTTTGGAATTAGTTAAGGAATGGAAGGAAAAGCCAGCTGGTGAATACGTTTCAGAAGAAGTTGAAATCTTGTTTACTGCAGTTTACAACACAGTTAACGAATTAGCTGAAATGGCACATATCGAACAAGGTAGATCTGTTAAGGATTTGTTGGTTAAGTTGTGGGTTGAAATCTTGTCAGTTTTTAGAATCGAATTGGATACTTGGACAAACGATACTGCTTTGACATTAGAAGAATATTTGTCTCAATCATGGGTTTCTATTGGTTGTAGAATCTGTATCTTGATCTCAATGCAATTCCAAGGTGTTAAGTTGTCTGATGAAATGTTGCAATCAGAAGAATGTACTGATTTGTGTAGATACGTTTCTATGGTTGATAGATTGTTGAACGATGTTCAAACTTTCGAAAAGGAAAGAAAGGAAAACACTGGTAATTCTGTTTCATTGTTACAAGCTGCACATAAGGATGAAAGAGTTATTAATGAAGAAGAAGCTTGTATCAAAGTTAAAGAATTGGCAGAATACAACAGAAGAAAGTTGATGCAAATTGTTTACAAGACTGGTACAATCTTCCCAAGAAAGTGTAAGGATTTGTTTTTAAAAGCTTGTAGAATCGGTTGTTATTTGTACTCTTCAGGTGACGAGTTTACTTCTCCACAACAAATGATGGAAGATATGAAGTCATTGGTTTACGAACCATTGCCAATCTCTCCACCAGAAGCTAATAATGCATCAGGTGAAAAGATGTCTTGTGTTTCAAATTAA |

The predicted N-terminal plastid transit peptide is marked in green.
